# Isolated Large Lateral Thoracic Meningocele

**DOI:** 10.7759/cureus.73041

**Published:** 2024-11-05

**Authors:** Sandisile M Ndlovu, Bindu Gaur, Arun Sureshkumar, Shouvik Saha, Reuben Sidhu

**Affiliations:** 1 Emergency Medicine, The Royal Wolverhampton NHS Trust, Wolverhampton, GBR; 2 Respiratory Medicine, The Royal Wolverhampton NHS Trust, Wolverhampton, GBR; 3 General Surgery, The Royal Wolverhampton NHS Trust, Wolverhampton, GBR; 4 Musculoskeletal Radiology, The Royal Wolverhampton NHS Trust, Wolverhampton, GBR; 5 Acute Internal Medicine, The Royal Wolverhampton NHS Trust, Wolverhampton, GBR

**Keywords:** conservative management, differential diagnoses, elderly patient, intrathoracic meningocele, isolated true meningocele, misdiagnosis, mri, pleural effusion misdiagnosis, spinal dural defects, thoracic meningocele

## Abstract

Intrathoracic meningocele is a condition characterized by the protrusion of dura mater and cerebrospinal fluid within the thoracic cavity. This can be associated with neurofibromatosis type 1 (NF1) and other connective tissue disorders. Less commonly, it can occur in isolation.

We describe one of the few cases of a large thoracic meningocele presenting in an adult without NF1 or another genetically predisposing condition. This case involves a 78-year-old woman who presented with back pain, shortness of breath, and lower limb weakness. The computed tomography (CT) scan reported a pleural effusion, with plans made to drain the fluid. However, magnetic resonance imaging (MRI) revealed a large right-sided lateral thoracic meningocele.

This case highlights the diagnostic challenges and potential complications associated with the misdiagnosis of thoracic meningoceles. It also reaffirms MRI as the gold standard for diagnosis.

## Introduction

A meningocele is defined as the protrusion of the dural sac containing cerebrospinal fluid (CSF) outside the spinal canal, typically through a bony defect. It is thought to arise from embryonic maldevelopment and can also be associated with genetic defects. The most common location for meningoceles is the lumbosacral region. Lateral thoracic meningoceles are uncommon, with reported cases in the hundreds [1]. When they do occur, 69% are associated with neurofibromatosis type 1 (NF1), and only 22% are isolated cases [1,2]. They may also be linked to connective tissue disorders such as Marfan syndrome. Patients may present with compression symptoms as the cyst enlarges, including pain and neurological deficits, or they may be asymptomatic, with the meningocele discovered incidentally. Thoracic meningoceles often present with respiratory symptoms, including dyspnea and cough, and can mimic COVID-19 infections [3].

In cases of lateral thoracic meningoceles without symptoms, surveillance is a recognized management plan. However, in patients with neurological or respiratory symptoms, surgery may be essential for symptom control [2].

In this case, we discuss an isolated case of true thoracic meningocele in a patient without known NF1 or any other connective tissue disorder. The patient presented with complications of the meningocele which was initially misdiagnosed as a pleural effusion. Although symptomatic, the patient did not undergo surgical management. 

This article was previously presented at the Manchester Medical Society of Imaging as an oral presentation on October 10, 2024. It was also presented as a poster presentation at the Society of Acute Medicine International conference on October 10, 2024.

## Case presentation

A 78-year-old woman presented to the emergency department (ED) with a one-week history of back pain and shortness of breath. She had a known history of sciatica but reported worsening pain over the last two weeks, which had significantly affected her mobility. Additionally, she mentioned a fall four weeks prior, attributing this to her back pain. On physical examination, she had reduced air entry on the right side of her chest and at the left lung base, generalized lumbar spine tenderness, and bilateral lower limb weakness. Her oxygen saturation was 89-90% on room air, improving to 95-96% on 3L of oxygen. Her medical history included a left mastectomy for breast cancer, sciatica, and frailty. Socially, the patient lived alone with no carers and was able to independently carry out activities of daily living.

Investigations

In the ED, the patient underwent routine blood tests and a chest radiograph. The blood tests revealed a raised white cell count (WCC) and C-reactive protein (CRP), with platelets within normal ranges (Table 1). 

**Table 1:** Laboratory workup, relevant results and ranges WCC: white cell count, CRP: C-reactive protein

Parameters	Value	Normal Range	Units
Hemoglobin	146	115 – 165	g/L
WCC	18.3	3.6 - 11.0	x10^9^/L
Platelet Count	359	140 - 400	x10^9^/L
CRP	202	<5	mg/L

**Figure 1:**
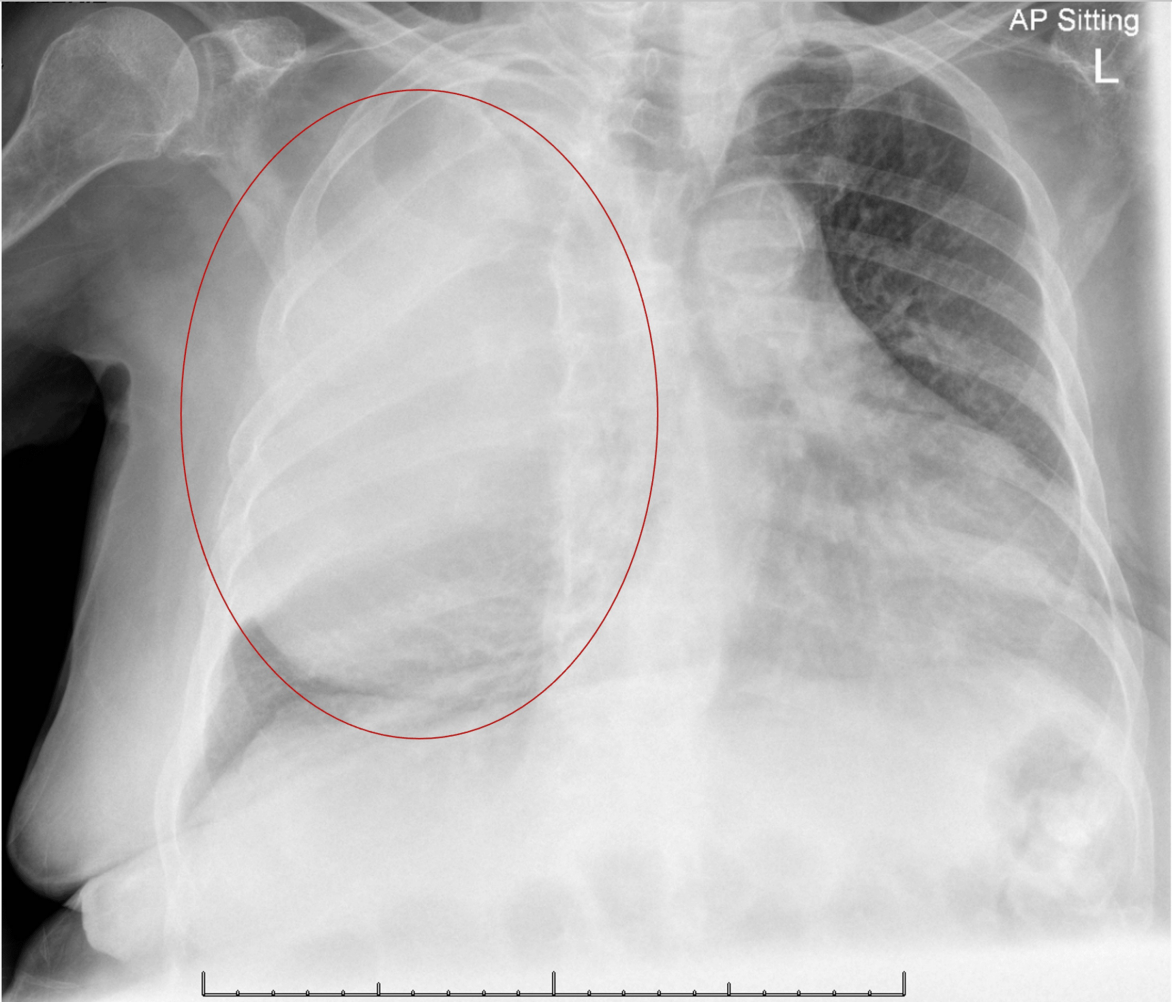
AP sitting chest X-ray AP: Anteroposterior

The chest radiograph showed near-complete opacification of the right hemithorax with mediastinal shift to the left (Figure 1), along with fractures of the right sixth and seventh posterolateral ribs.

**Figure 2:**
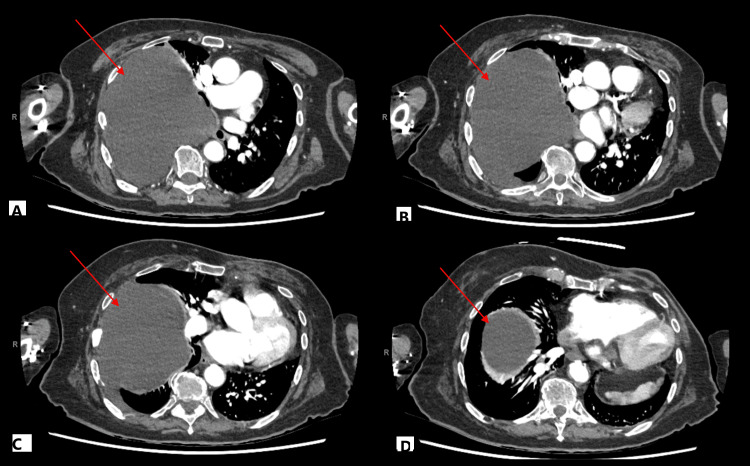
Selected axial CT images of the thorax

Given these findings, a computer tomography scan of the thorax, abdomen, and pelvis (CT TAP) was performed (Figure 2). The reporting radiology registrar described the mass as a large loculated pleural effusion with mediastinal deviation. However, the consultant’s addendum identified a large cyst emerging from the right T4 intervertebral foramen, splaying the fourth and fifth ribs. Chronic rib fractures were noted alongside acute fractures of the pubic rami, sacral alar, and L5 transverse process.

**Figure 3:**
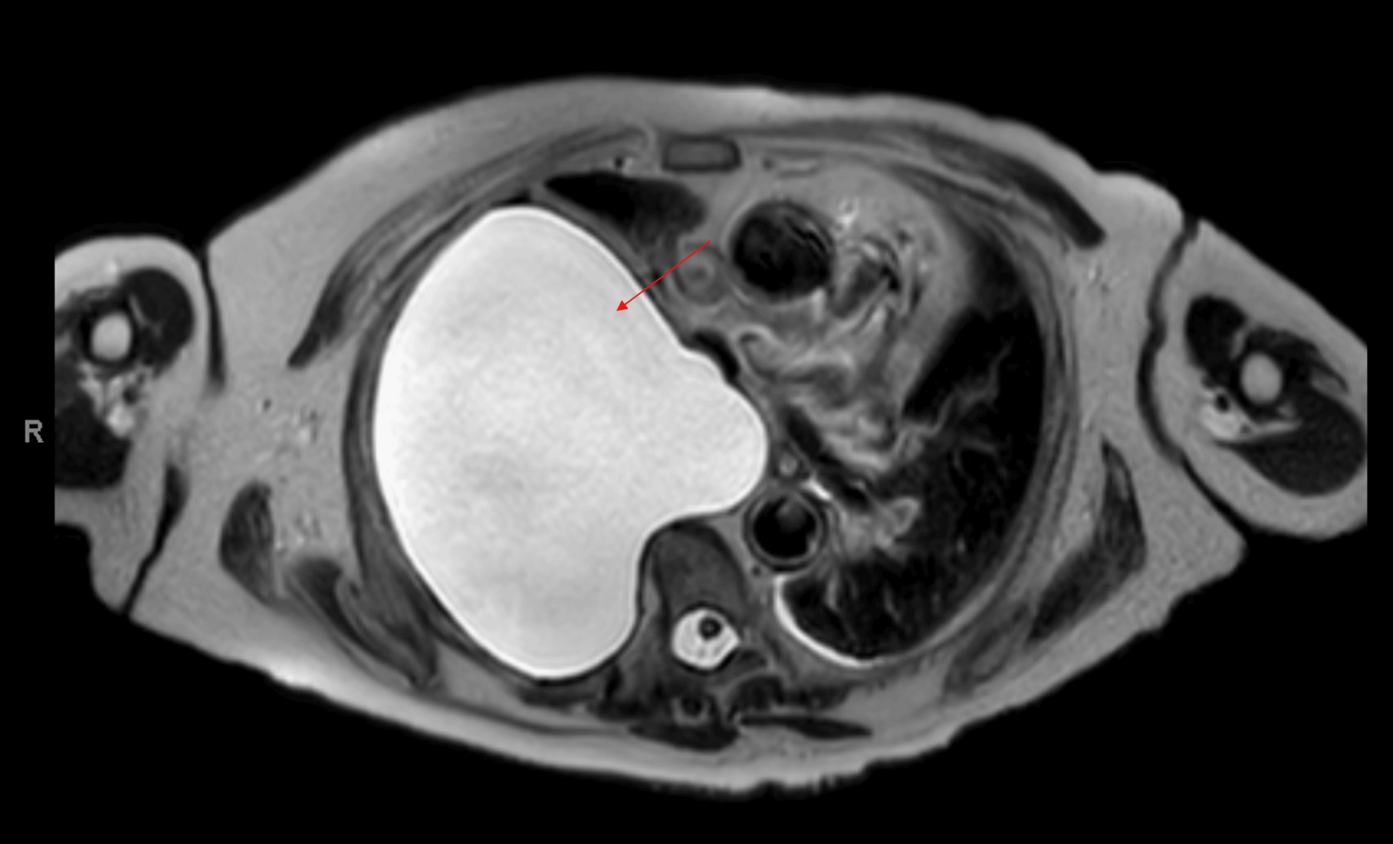
Axial T2 weighted MRI image

**Figure 4:**
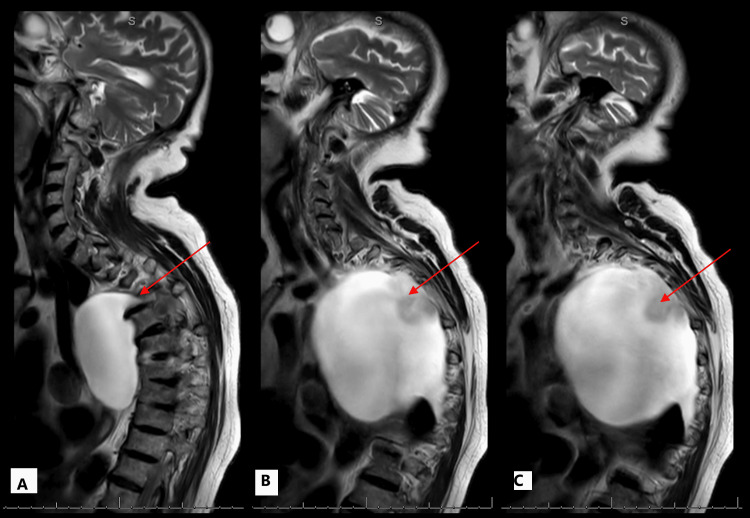
Sagittal T2 weighted MRI images

Further imaging with MRI as an inpatient revealed a large right-sided lateral thoracic meningocele arising from the T4-5 foramen (Figure 3 and Figure 4). A small right pleural effusion was also noted, but there was no evidence of neural impingement or cord or cauda equina compromise.

Management

The patient was initially managed in the ED with broad-spectrum antibiotics for a suspected respiratory infection. On the first night of admission, her condition deteriorated, triggering a sepsis alert. Discussions were held regarding whether to drain what was initially thought to be a pleural effusion based on the CT report. However, the patient was managed conservatively with intravenous fluids and oxygen therapy.

Following the MRI, which confirmed the diagnosis of a thoracic meningocele, the patient was referred to a tertiary center for discussion at a multidisciplinary team (MDT) meeting with neurosurgery. The MDT recommended outpatient follow-up after discharge to assess further management, including evaluating the patient's suitability for surgical options. However, her functional status continued to decline during hospitalization. It is suspected that the combination of infection and the meningocele contributed to the deterioration from which the patient did not recover. She experienced reduced oral intake, decreased activity levels, and a continued need for oxygen. Ultimately, she was transitioned to supportive care, and anticipatory medications were initiated for palliative care. She was transferred to a hospice, where she passed away weeks later. The cause of death was recorded as pneumonia, with the thoracic meningocele as a contributing factor.

## Discussion

Our patient was not found to have NF1, Marfan syndrome, or lateral meningocele syndrome - all hereditary conditions associated with intrathoracic meningoceles - nor was there a family history of these conditions. This therefore represents a truly isolated presentation. Intrathoracic meningoceles are reported to occur in NF1 in 60-85% of cases [4], making their occurrence outside of this context significantly rarer, as reflected by the lack of recent literature in the past 20 years. The most recent documented cases of isolated meningoceles have been in children with spinal dysraphism, such as congenital scoliosis [1,5,6,7]. 

In the absence of NF1, thoracic meningoceles may be associated with genetic defects or embryonic maldevelopment, presenting as congenital bone defects, thus making the individual susceptible to its development during their life [8]. Although the precise developmental mechanisms leading to thoracic meningoceles remain speculative, several contributing factors have been suggested. These include congenital herniation of the subarachnoid space, elongation of the nerve root sheath, dural dysplasia, spinal dysplasia, and cystic changes in neurofibromas [7,8]; the latter only applies to intrathoracic meningoceles in NF1 patients. Maiuri et al. propose that spinal trauma may also play a role in their development [9]. Given our patient’s advanced age and recent fall, it is plausible that spinal trauma contributed to meningocele formation.

When intrathoracic meningoceles appear in adulthood, they are typically found in patients aged 30-60 years [10]. This case is unusual as the patient presented in old age, 78, the oldest documented in the literature review. It is unclear if the meningocele was present from birth and went undetected or developed later in life. Previous radiological images from another NHS trust, which were unavailable, might have helped determine how long the meningocele had been present or if there was any spinal dysplasia present prior to the meningocele formation.

In this case, the patient presented with respiratory complications from her large thoracic meningocele and was initially misdiagnosed as having a pleural effusion. There has been a previously documented case in which a meningocele was misdiagnosed as a pleural effusion, and the fluid drained [11]. The drainage improved the patient’s respiratory symptoms but resulted in the patient developing a severe postural headache, similar to that caused post-lumbar puncture. It also puts the patient at risk of complications of drainage, such as meningitis or pneumocephalus. Fortunately, the plans for this patient to drain the fluid were delayed until further imaging could be obtained, which confirmed a meningocele. Other documented complications of meningoceles include spontaneous subarachnoid pleural fistula, which can occur when a meningocele ruptures into the pleural space, presenting with severe headaches and breathing difficulties [12].

Management of thoracic meningoceles depends on the size and location of the meningocele and the presence of symptoms. Peritoneal shunts, such as cystoperitoneal or ventriculoperitoneal shunts, are less invasive techniques for draining CSF from the meningocele into the peritoneal cavity, alleviating symptoms [13,14]. A cystoperitoneal shunt can be placed under local anesthesia [15]. However, they are not always effective in treating giant intrathoracic meningoceles, and surgery may be required for a definitive outcome [16]. A more invasive option is thoracotomy, which provides a broader operative field to drain the cyst and repair the dura mater, particularly for large thoracic meningoceles [17]. Surgery carries risks, including bleeding, infection, and recurrence [2]. In this case, surgery was deemed high risk during the acute phase, and the MDT recommended reassessment if the patient recovered from the acute episode. Had the patient recovered, a full evaluation of cardiorespiratory function, comorbidities, and frailty would have been necessary to assess her physiological reserve and suitability for surgery.

## Conclusions

In summary, this case highlights the diagnosis and conservative management of an intrathoracic meningocele presenting in adulthood without known NF1. Although the incidence is low, there have been hundreds of documented cases. As CT scans are typically the first line of investigation, thoracic meningoceles should be considered in the differential diagnosis when reviewing large fluid collections in patients presenting with respiratory and neurological symptoms. This should prompt further investigation with MRI, which is the gold standard to avoid misdiagnosis. Early detection can help prevent unnecessary and potentially harmful procedures that may result in iatrogenic complications. Interdisciplinary collaboration is essential in these cases, with early involvement of neurosurgery to develop a holistic care plan and assess suitability for surgical intervention, especially for elderly patients with comorbidities.

## References

[1] Oner AY, Uzun M, Tokgöz N, Tali ET (2004). Isolated true anterior thoracic meningocele. AJNR Am J Neuroradiol.

[2] Elsayed AA, Rajabian A, Nabi A, Du Plessis D, George KJ (2022). Thoracic meningocele in patients with neurofibromatosis type 1: a review of literature with illustration of a novel surgical challenge, and insights from histology. Interdiscip Neurosurg.

[3] Mousafeiris VK, Papaioannou I, Pantazidou G, Kalyva N, Repantis T (2022). An intrathoracic meningocele in a neurofibromatosis type I patient mimicking severe COVID-19 disease. Cureus.

[4] Hirbe AC, Gutmann DH (2014). Neurofibromatosis type 1: a multidisciplinary approach to care. Lancet Neurol.

[5] Ran J, Karamian P, Robinow Z, Lui F, Gonda D (2023). Anterolateral thoracic myelomeningocele with split cord malformation. Cureus.

[6] Loukili H, Outaghyame S, Ahmanna C, Zouita B, Basraoui D, Jalal H (2024). A rare case of an isolated intra-thoracic meningocele: case report and review of the literature. Sch J Med Case Rep.

[7] Turgut M, Alhan C, Cihangiroglu M, Topcuoglu MS (2004). Isolated giant intrathoracic meningocele associated with vertebral corpus deformity. Interact Cardiovasc Thorac Surg.

[8] Miles J, Pennybacker J, Sheldon P (1969). Intrathoracic meningocele. Its development and association with neurofibromatosis. J Neurol Neurosurg Psychiatry.

[9] Maiuri F, Corriero G, Giampaglia F, Simonetti L (1986). Lateral thoracic meningocele. Surg Neurol.

[10] Geerts Y, Marchau M (1992). Intrathoracic meningocele. J Spinal Disord.

[11] Chen N, Li W, Min L, Huang Q, Bian J (2024). Neurofibromatosis type 1 with huge intrathoracic meningoceles misdiagnosed as pleural effusion: a case report and literature review. J Cardiothorac Surg.

[12] Kumar V, Bundela YS, Gupta V, Dua S, Singh AK (2010). Spontaneous subarachnoid pleural fistula: a rare complication of lateral thoracic meningocele. Neurol India.

[13] Vanhauwaert DJ, Deruytter MJ (2008). Cystoperitoneal shunt as alternative treatment for a giant thoracic meningocele in a patient with neurofibromatosis. Surg Neurol.

[14] Yurter A, Kaloostian PE (2013). Giant thoracic meningocele causing acute respiratory compromise. J Modern Neurosurg.

[15] Tanaka K, Shimizu K, Kakegawa S, Oshima K, Takeyoshi I (2011). Cystoperitoneal shunt for a giant intrathoracic meningocele under local anesthesia. Ann Thorac Surg.

[16] Sum CH, Li LF, Taw BB, Lui WM, Sit KY, Chow VL, Wong YW (2021). Surgical repair of a large intrathoracic meningocele associated with neurofibromatosis type 1 after failed cystoperitoneal shunts: illustrative case. J Neurosurg Case Lessons.

[17] Kim YJ, Cho HM, Yoon CS, Lee CK, Lee TY, Seok JP (2011). Surgical treatment of thoracic menigocele associated with neurofibromatosis and kyphoscoliosis. Korean J Thorac Cardiovasc Surg.

